# Identifying current and remitted major depressive disorder with the Hurst exponent: a comparative study on two automated anatomical labeling atlases

**DOI:** 10.18632/oncotarget.19860

**Published:** 2017-08-03

**Authors:** Bin Jing, Zhuqing Long, Han Liu, Huagang Yan, Jianxin Dong, Xiao Mo, Dan Li, Chunhong Liu, Haiyun Li

**Affiliations:** ^1^ School of Biomedical Engineering, Capital Medical University, Beijing, China; ^2^ School of Software Engineering, Beijing University of Technology, Beijing, China; ^3^ Acupuncture and Moxibustion Department, Beijing Hospital of Traditional Chinese Medicine Affiliated to Capital Medical University, Beijing, China; ^4^ Beijing Key Laboratory of Mental Disorders, Department of Radiology, Beijing Anding Hospital, Capital Medical University, Beijing China

**Keywords:** hurst exponent, automated anatomical labeling (AAL) atlas, resting-state fMRI, support vector machine, major depressive disorder

## Abstract

Major depressive disorder (MDD) is a leading world-wide psychiatric disorder with high recurrence rate, therefore, it is desirable to identify current MDD (cMDD) and remitted MDD (rMDD) for their appropriate therapeutic interventions. In the study, 19 cMDD, 19 rMDD and 19 well-matched healthy controls (HC) were enrolled and scanned with the resting-state functional magnetic resonance imaging (rs-fMRI). The Hurst exponent (HE) of rs-fMRI in AAL-90 and AAL-1024 atlases were calculated and compared between groups. Then, a radial basis function (RBF) based support vector machine was proposed to identify every pair of the cMDD, rMDD and HC groups using the abnormal HE features, and a leave-one-out cross-validation was used to evaluate the classification performance. Applying the proposed method with AAL-1024 and AAL-90 atlas respectively, 87% and 84% subjects were correctly identified between cMDD and HC, 84% and 71% between rMDD and HC, and 89% and 74% between cMDD and rMDD. Our results indicated that the HE was an effective feature to distinguish cMDD and rMDD from HC, and the recognition performances with AAL-1024 parcellation were better than that with the conventional AAL-90 parcellation.

## INTRODUCTION

Major depressive disorder (MDD) is a common psychiatric disease, which is characterized by a low mood, worthlessness, anxiety and cognitive impairments [1, 2], and these symptoms are associated with the structural and functional impairments of some core brain regions [3]. By the year 2020, it will be the second leading global disease burden [4]. Besides, remitted MDD (rMDD) represents a not full recovery state of MDD, which also shows a tremendous effect on outcomes such as future relapse, morbidity and mortality [5]. Currently, no effective neurobiological markers or predictors are adopted to confirm the current MDD (cMDD) and rMDD in clinical practice, and the diagnosis of cMDD and rMDD are mainly based on the clinical symptoms and signs, and expert consensus. So it is desirable to develop effective brain imaging based diagnostic methods to identify cMDD and rMDD from healthy controls (HC), which could provide an objective perspective to recognize and understand the cMDD and rMDD.

Magnetic resonance imaging (MRI) has attracted increasing attention for improving our understanding of the pathological mechanism and the underlying cognitive and affective dysfunctions in MDD [6]. A consistent finding is that MDD patients showed functional and structural abnormalities in limbic areas, prefrontal cortical regions and subcortical structures relative to HC [7–9], and the abnormalities in these brain regions may reflect the physiological characteristics of depressive patients. However, few studies have investigated the resting state brain abnormalities in rMDD patients. Yuan et al. reported that the remitted geriatric depression patients showed increased regional homogeneity (ReHo) in right median frontal gyrus, superior frontal gyrus and putamen, and decreased ReHo in parietal and temporal gyrus [10]. In our previous study, we found increased amplitude of low-frequency fluctuation (ALFF)/fractional ALFF (fALFF) values in right putamen, and decreased ALFF/fALFF values in right precuneus and left lingual gyrus in remitted depression patients [11]. Though great progress has been made in cMDD and rMDD studies during the past decades, patient-specific diagnostic methods for cMDD and rMDD are still desperately needed.

In recent years, machine learning has been applied to identify MDD patients based on MRI signals. Fu et al. classified MDD patients from HC on the basis of their neural responses to the experiments of sad faces presentation, and accuracies of 74% and 76% were obtained with medium-intensity sad faces and high-intensity sad faces respectively [1]. Hahn et al. used a Gaussian process classifier to identify MDD patients based on the integrating functional MRI (fMRI) data associated with affective and emotional processing, and acquired an accuracy of 83% [12]. Wei et al. adopted the Hurst exponent (HE) of twelve resting-state fMRI networks as the classification features, and achieved an accuracy of 90% [13, 14]. Marquand et al. investigated the functional neuroanatomy of verbal working memory as a potential diagnostic biomarker for depressive disorders, and got an accuracy rate of 68% [15]. Costafreda et al. used the support vector machine (SVM) algorithm on anatomical MRI data of MDD patients with pharmacological treatment, and yielded an accuracy of 89% [16]. To the best of our knowledge, few studies have utilized the machine learning method to discriminate every pair of the cMDD, rMDD and HC groups.

Appropriate brain parcellations have become a pursuing goal since the widespread applications of multi-modal MRI imaging [17–20], and different scaling atlases have been reported to result in considerable variations in relative studies [21, 22]. Among the existing atlases, the Automated Anatomical Labeling (AAL-90) atlas is still the most popular atlas in the brain studies [23], and it has been widely used in the discriminative studies for different disorders [24–26]. Notably, some previous studies demonstrated that the structural-MRI characteristics based recognition rates were affected by the selection of brain atlases [27, 28]. However, to the best of our knowledge, few studies have explored whether the fMRI characteristics (e.g. HE) based identification accuracies were also dependent on the atlas choice. In this study, AAL-1024 atlas was selected to compare with the conventional AAL-90 atlas for the following reasons. First, unlike other atlases, the AAL-1024 atlas is generated from the AAL-90 atlas, so it is easy to interpret and compare the abnormal regions between these two atlases. Second, the AAL-1024 atlas has 1024 subregions with identical size (approximate 40 voxels), so the influences of different sizes of subregions are avoidable. Moreover, considering some regions in the AAL-90 atlas are large, it is most likely that the signals from these regions are derived from several different functional subregions, which may influence the between-group findings, but the AAL-1024 atlas may better overcome this shortage.

Existing studies have demonstrated that spontaneous brain activities display scale-free dynamics, suggesting that the resting-state blood oxygen level-dependent (BOLD) signals show fractal-like properties [29]. HE, as an index ranging from 0 to 1, could well display the scale-free dynamics via describing the self-similarity of a time series [30, 31]. A HE bigger than 0.5 indicates a positively correlated or persistent behavior in the time series, and a HE smaller than 0.5 implies an anti-correlated time series, i.e., a decrease in time series will be generally followed by an increase in time series, while a HE equals to 0.5 indicating a random white-noise time series [32]. Recently, HE has been utilized widely to access pathological and physiological conditions. An increasing HE was reported to accompany with normal aging in bilateral hippocampus [33]. Besides, changes in HE were shown to be associated with autism spectrum disorders, cholinergic modulation and different personality traits [34–36]. However, the HE differences among cMDD patients, rMDD patients and HC groups still remain unknown, and it is also uncertain whether HE could be an effective feature to discriminate every pair of the cMDD patients, rMDD patients and HC groups.

In this paper, a radial basis function (RBF) based SVM method was proposed to identify every pair of cMDD, rMDD and HC groups using HE index. First, the HE characteristics of three groups were calculated by means of a rescaled range (R/S) analysis, and then the mean HE values in two AAL atlases (AAL-1024 and AAL-90) were computed and compared between groups. At last, the mean HE values of abnormal brain regions were served as classification features of the RBF based SVM algorithm to discriminate every pair of the three groups, and a leave-one-out cross-validation (LOOCV) was applied to evaluate the recognition performance.

## RESULTS

### Demographic and clinical information

Table 1 summarized the demographic and clinical information about the cMDD patients, rMDD patients and HC groups. There were significant differences in Hamilton Depression Rating Scale (HAMD) scores between the cMDD and rMDD groups, but not in duration of illness and the number of episodes. No significant differences were observed in age and education level among three groups.

**Table 1:** Participant demographic and clinical characteristics

Variables (Mean ± SD)	cMDD	rMDD	HC	*P* Values
Number of subjects	19	19	19	—
Age (years)	34.84 ± 13.58	37.58 ± 12.69	36.84 ± 12.69	0.79^a^
Education (years)	12.84 ± 3.18	12.68 ± 2.93	13.74 ± 2.16	0.46^a^
Duration of illness (years)	6.90 ± 8.34	7.37 ± 5.53		0.84^b^
HAMD	21.65 ± 4.50	4.63 ± 2.57		0.00^b^
Number of depressive episodes	2.68 ± 1.95	2.39 ± 1.80		0.64^b^
Antidepressants	23	29		
Mood-stabilizer	1	1		
Antipsychotics	4	6		
Benzodiazepines	2	1		
Medication-free	3	1		

SD: standard deviation; cMDD: current major depressive disorder; rMDD: remitted major depressive disorder; HC: healthy controls; HAMD: Hamilton Depression Rating Scale.

^a^The *P* values were obtained by one-way ANOVA.

^b^The *P* values were obtained by two-sample two-tailed *t*-tests.

### Classification performance with two AAL atlases

**Figure 1:**
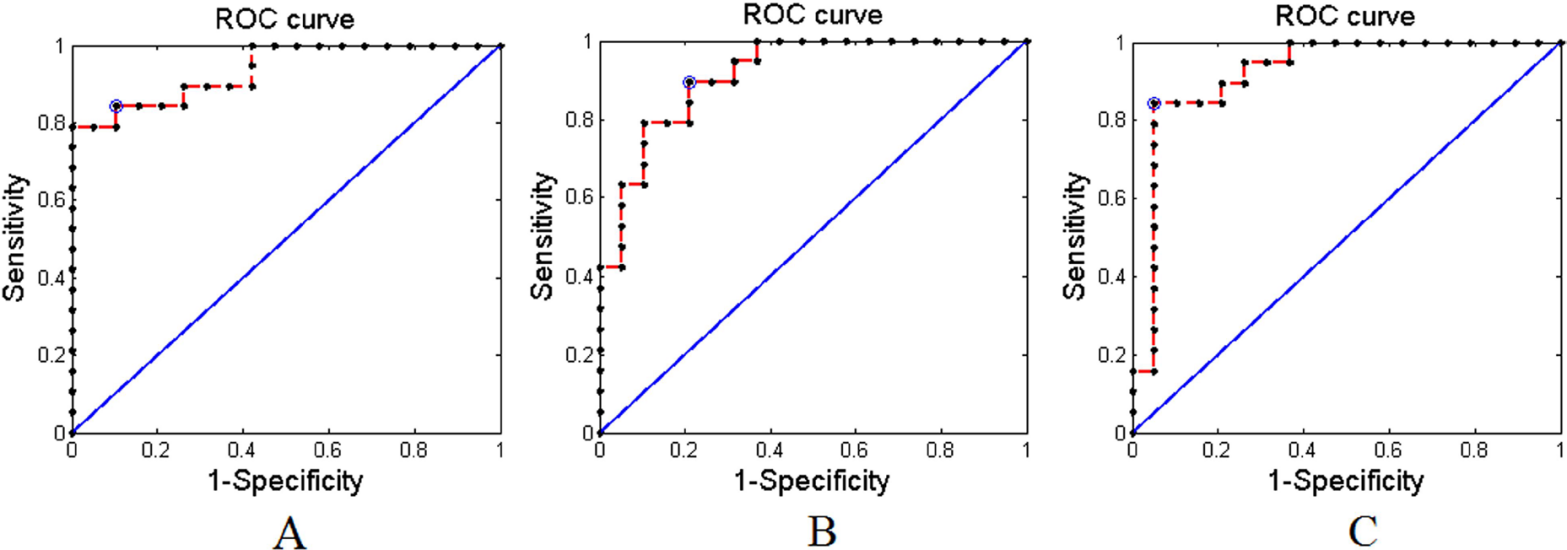
The receiver operating characteristics (ROC) curves of the proposed method with AAL-1024 atlas (**A**) Between cMDD patients and HC; (**B**) Between rMDD patients and HC; (**C**) Between rMDD patients and cMDD patients.

**Figure 2:**
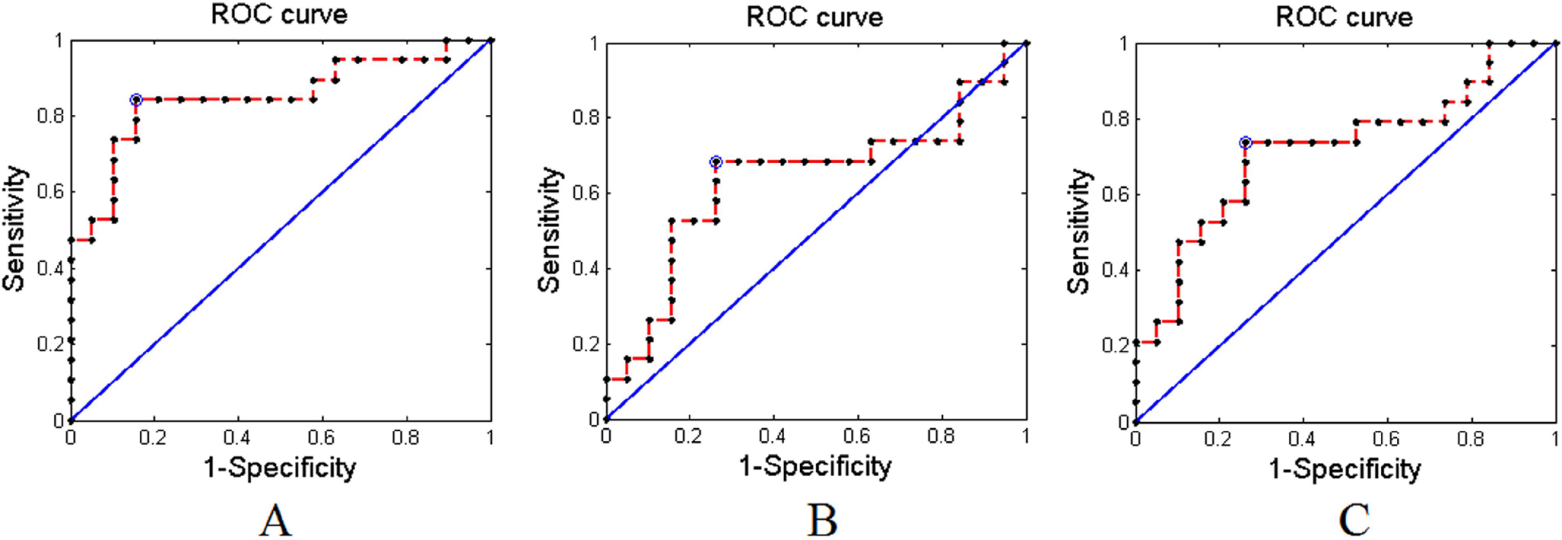
The receiver operating characteristics (ROC) curves of the proposed method with AAL-90 atlas (**A**) Between cMDD patients and HC; (**B**) Between rMDD patients and HC; (**C**) Between rMDD patients and cMDD patients.

**Figure 3:**
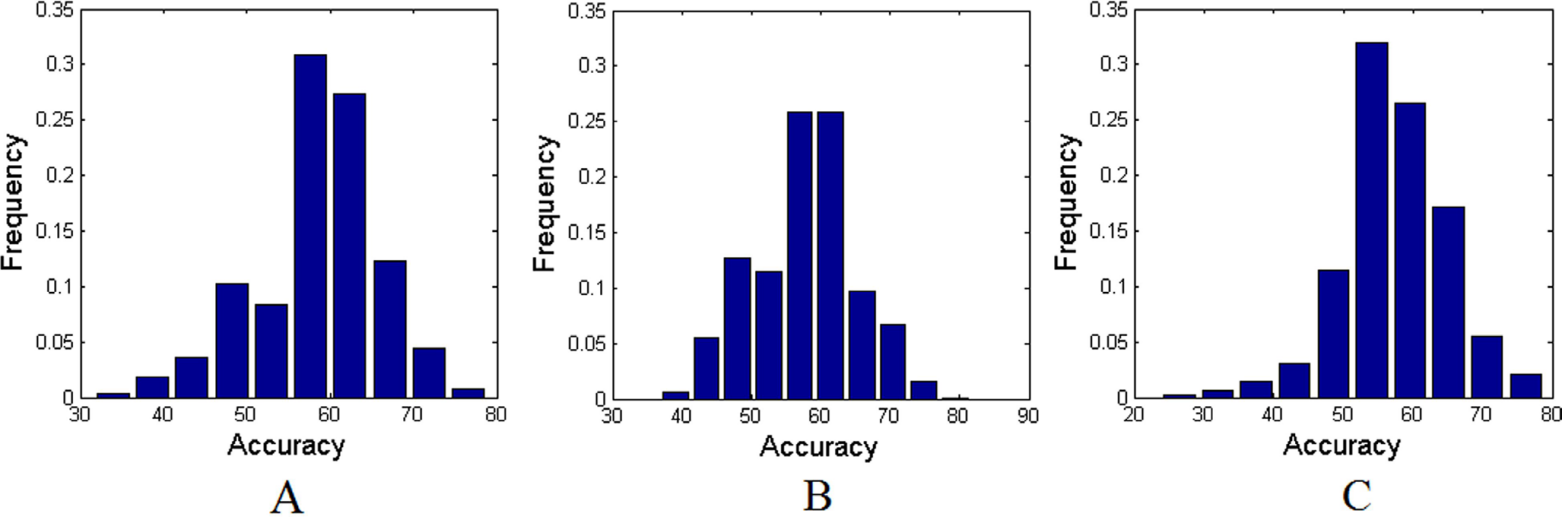
The permutation distributions of accuracies with AAL-1024 atlas (**A**) Between cMDD patients and HC; (**B**) Between rMDD patients and HC; (**C**) Between rMDD patients and cMDD patients.

**Figure 4:**
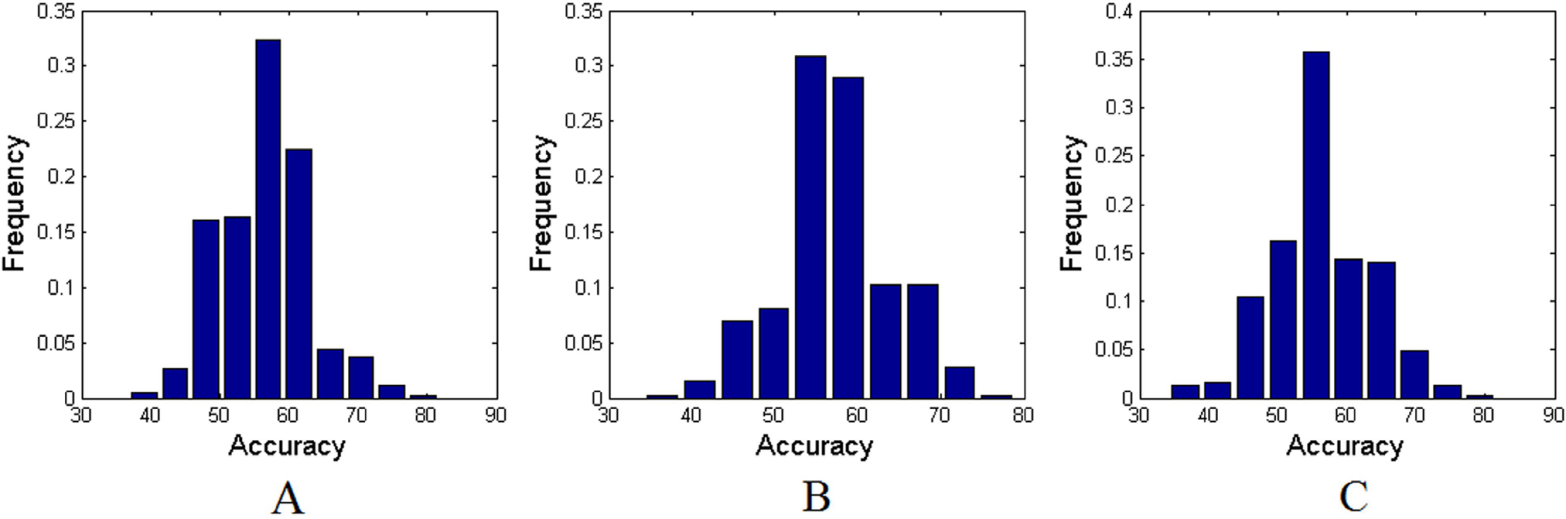
The permutation distributions of accuracies with AAL-90 atlas (**A**) Between cMDD patients and HC; (**B**) Between rMDD patients and HC; (**C**) Between rMDD patients and cMDD patients.

Applying the proposed method to the experimental MRI data with AAL-1024 atlas, 87% of the subjects were correctly classified between cMDD patients and HC, together with an accuracy of 84% between rMDD patients and HC, and an accuracy of 89% between cMDD patients and rMDD patients. The detailed results were listed in Table 2. Taking every subject's predicted score as a threshold, the corresponding receiver operating characteristics (ROC) curves were acquired as shown in Figure 1, and the area under the ROC curves (AUCs) of the proposed method were 0.94, 0.91 and 0.92, respectively. Table 3 listed the number of features retained in the proposed method per fold. Utilizing the proposed method to the experimental MRI data with AAL-90 atlas, 84% of the subjects were correctly classified between cMDD patients and HC, together with an accuracy of 71% between rMDD patients and HC, and an accuracy of 74% between cMDD patients and rMDD patients. Table 4 summarized the detailed results, and three ROC curves were also acquired as shown in Figure 2, and the AUCs of the proposed method were 0.85, 0.64 and 0.72, respectively. The number of features retained in the proposed method per fold was showed in Table 5. Besides, all *P* values of the permutation test were less than 0.01, indicating the classification accuracies were reliable, and the relevant statistical results were showed in Figure 3 and Figure 4.

**Table 2:** The classification performance in every pair of cMDD, rMDD and HC with AAL-1024 atlas

Groups	Accuracy	Sensitivity	Specificity
cMDD vs. HC	87%	84%	89%
rMDD vs. HC	84%	89%	79%
cMDD vs. rMDD	89%	84%	95%

**Table 3:** The number of features retained in the proposed method per fold with AAL-1024 atlas

Fold	Between cMDD and HC	Between rMDD and HC	Between cMDD and rMDD
1	75	73	63
2	72	84	73
3	78	76	70
4	82	84	68
5	73	71	74
6	71	64	65
7	86	89	71
8	72	64	63
8	75	70	66
10	67	70	64
11	71	69	69
12	66	86	66
13	71	71	59
14	76	78	61
15	76	64	61
16	74	71	65
17	69	68	57
18	74	73	67
19	74	81	62
20	82	78	66
21	79	79	70
22	77	82	68
23	78	66	62
24	73	76	58
25	69	73	67
26	92	78	66
27	73	67	69
28	65	82	65
29	69	80	57
30	76	82	58
31	80	69	69
32	73	85	73
33	87	62	63
34	62	74	58
35	71	75	63
36	73	68	65
37	86	67	63
38	83	66	61

**Table 4:** The classification performance in every pair of cMDD, rMDD and HC with AAL-90 atlas

Groups	Accuracy	Sensitivity	Specificity
cMDD vs. HC	84%	84%	84%
rMDD vs. HC	71%	68%	74%
cMDD vs. rMDD	74%	74%	74%

**Table 5:** The number of features retained in the proposed method per fold with AAL-90 atlas

Fold	Between cMDD and HC	Between rMDD and HC	Between cMDD and rMDD
1	8	5	3
2	9	7	3
3	9	5	5
4	7	6	5
5	7	7	4
6	8	3	4
7	11	8	6
8	8	4	4
8	8	5	6
10	8	3	4
11	8	5	7
12	9	7	5
13	8	5	3
14	10	7	5
15	8	4	3
16	8	4	5
17	10	7	3
18	7	4	4
19	7	6	6
20	9	5	6
21	9	9	4
22	8	5	4
23	8	4	3
24	9	5	3
25	8	3	4
26	7	3	4
27	10	7	5
28	8	9	4
29	8	6	4
30	9	6	3
31	9	6	4
32	8	8	8
33	8	3	6
34	7	4	3
35	8	4	6
36	8	5	5
37	9	3	4
38	8	3	5

### Between-group differences in HE

**Figure 5:**
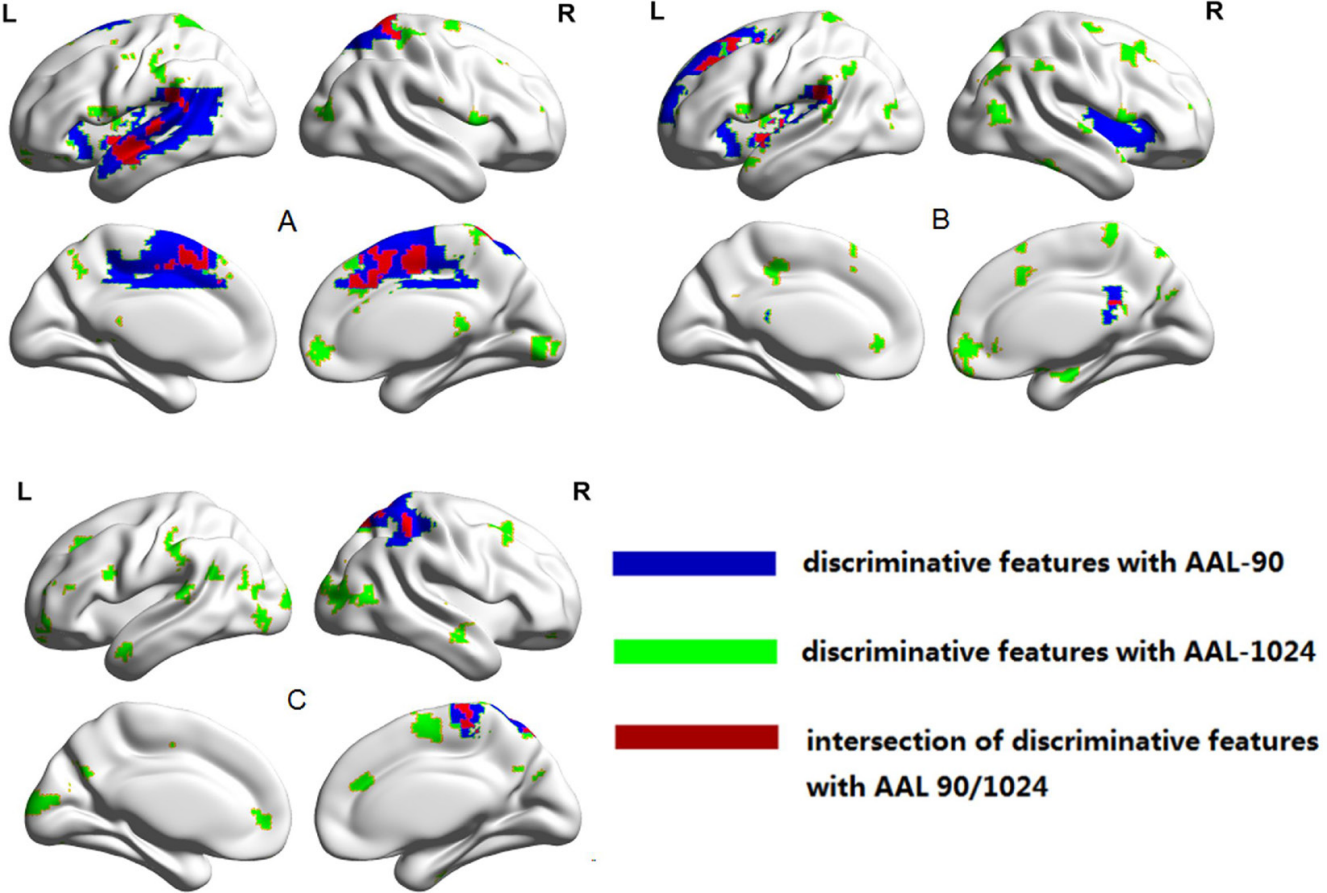
The HE differences in every pair of cMDD patients and rMDD patients and HC (**A**) Between cMDD patients and HC; (**B**) Between rMDD patients and HC; (**C**) Between rMDD patients and cMDD patients.

The most discriminative brain regions for group separation with AAL-1024 atlas and AAL-90 atlas were showed in Figure 5. When classifying cMDD patients from HC, the most informative regions mainly contained left insula, bilateral cingulate gyrus, left middle temporal gyrus, left superior temporal gyrus, bilateral supplementary motor area and right superior parietal cortex. When classifying rMDD patients from HC, the brain regions with great discriminative power mostly included bilateral insula, right cingulate gyrus, left superior frontal gyrus and left superior temporal gyrus. While applying the proposed method to discriminate between cMDD patients and rMDD patients, the most informative brain regions were predominantly located in left middle frontal gyrus, bilateral middle occipital gyrus, right superior parietal cortex, and right inferior parietal lobule.

## DISCUSSION

In this paper, a RBF based SVM algorithm was proposed to discriminate every pair of the cMDD patients, rMDD patients and HC groups using the HE of resting-state fMRI. Compared with the AAL-90 atlas, the proposed method with AAL-1024 atlas obtained better recognition performances, and yielded an accuracy of 87% between cMDD patients and HC, 84% accuracy between rMDD patients and HC and 89% accuracy between cMDD patients and rMDD patients.

The proposed classification method has several advantages. First, the RBF kernel function is simple (less parameters), and it has few numerical problems (0 < *K*(*X, X_i_*) < 1). Unlike linear kernel function, it can deal with the case when the relationship between features and labels is nonlinear [37]. Second, a grid search algorithm, which has a high learning accuracy and can be implemented with parallel processing, was utilized to optimize the two parameters of SVM, and it significantly improved the classification performance. Third, two-sample two-tailed t tests were used to select out the discriminative features, which also has an important impact on the final performance. Several existing studies have demonstrated that correctly reducing the number of features can not only improve the classification performance but also speed up the computation [38, 39]. Meanwhile, considering the feature selection process was only constrained on the training set of each LOOCV fold, it could reduce overfitting of the classifier [24]. Besides, the total 90 and 1024 HE classification features were also tested using our proposed method respectively, and the classification accuracies without feature selection between any two groups were lower than 70%, which were notably lower than that with feature selection. In addition, the split-half validation, which divided every pair of cMDD, rMDD and HC into two groups, one group for training (9 subjects in each category) and one group for testing (10 subjects in each category), and the model generated by the training set were used to classify the testing set. With AAL-90 and AAL-1024 atlas respectively, we obtained accuracies of 70% and 80% between cMDD and HCs, 80% and 90% between rMDD and HCs, 70% and 85% between cMDD and rMDD. At last, considering that using a well-diagnosed but heterogeneous group of patients with different severity levels of clinical symptoms while not excluding medicated patients could offer a more realistic estimation of the proposed classification method for MDD [12], therefore, we combined the cMDD and rMDD patients as one group, and then discriminated the new combined group from HC. An accuracy of 75% was obtained using AAL-90 atlas, and 81% accuracy was achieved utilizing the AAL-1024 atlas, which reflected that our HE-based recognition method could effectively discriminate most MDD patients with heterogeneous symptoms and medication.

HE has become a widely used complexity index in recent resting-state fMRI studies. For example, it had been used as the diagnostic biomarkers for Alzheimer's disease [30], Autism Spectrum Disorders and Schizophrenia [34, 40]. These studies implied the possibility of HE as an independent discriminative feature in other psychiatric diseases. In a previous study, the HE was used to recognize the MDD patients from HC [13], and the mean HE values of twelve independent networks were selected as the discriminative features, but some networks (e.g. auditory, lateral visual network) that may be unrelated to MDD symptoms showed large weights among all features in the discrimination, which made the recognition rate seem not sound enough. By using the AAL-1024 atlas and AAL-90 atlas, our proposed method could find the more precise between-group features than the independent networks, and the reported regions may be more closely correlated with the MDD. Additionally, the physiological significance of HE is still uncertain, and it is hard to interpret the between-group abnormalities definitely. Future studies should be conducted to confirm the physiological significance of HE through the multi-modal imaging validations in the animal model.

In this paper, we separately discriminated every pair of cMDD patients, rMDD patients and HC groups with two AAL atlases, and found that the classification performance with AAL-1024 atlas was significantly better than that with AAL-90 atlas, which may arise from the differences in the discriminative features. To clearly elucidate the differences of the discriminative features between AAL-1024 and AAL-90 atlas, three tables were added in the Supplementary Tables 1, 2, and 3 to list all the stable discriminative features in LOOCV for the between-group classifications. From the tables, two advantages could be found with AAL-1024 atlas: First, all abnormal regions reported by AAL-90 were also detected by AAL-1024, and the discriminative features could be further located into more precise position by AAL-1024 atlas. For example, AAL-1024 atlas could discover more than one abnormal subregion in the regions reported by AAL-90, and this may be attributed to the existence of functional heterogeneity in AAL-90 parcellation, especially for some large regions. Second, AAL-1024 atlas could also detect some abnormal regions overlooked by AAL-90 atlas, therefore, more discriminative features were obtained by AAL-1024 than AAL-90. Moreover, these new features discovered by AAL-1024 mainly lied in the default mode network and the limbic system, which highly correlated with the pathology of major depressive disorder [11, 41, 42]. In addition, to further display the advantages of AAL-1024 atlas, the Fisher score method [43] was applied to all candidate features to detect whether the features revealed by AAL-1024 atlas could provide more discriminative power over the AAL-90 atlas. To have a comprehensive and direct comparison, the rearranged 90 features from AAL-90 atlas and the prior 90 features from AAL-1024 atlas were compared in Fisher score. From Figure 1 and Supplementary Figure 1 in Supplementary Material, the Fisher score of AAL-1024 was found significant larger than that of AAL-90, which again indicated the advantage of AAL-1024 atlas over AAL-90 atlas. Hurst exponent is a functional index for resting state fMRI, but the widely used AAL-90 atlas is generated based on anatomical information, therefore, AAL-90 atlas may be not sensitive to the functional abnormalities in local regions. Although AAL-1024 atlas is also not a functional brain atlas, it partitions the whole brain into 1024 subdivisions with relative small size, therefore, it could largely improve the sensitivity to the local functional anomaly but also incorporate the local anatomical information.

In this study, the abnormal regions detected by the HE analysis in cMDD and rMDD patients turned out to be informative. Compared with HC, both cMDD and rMDD patients showed abnormal HE values in left insula, right cingulate gyrus and left superior temporal gyrus, meaning that these brain regions may be the trait markers for MDD. The insula plays an important role in affective processing, and several studies found that the induced anxiety, retardation and agitation were associated with the insula [2, 44, 45]. The abnormality pattern in the left insula was also consistent with our previous voxel-based morphometry (VBM) study, which reported that the left dorsal anterior insula volume was found atrophy in both cMDD and rMDD groups [42]. The cingulate gyrus is commonly considered as a critical node of default mode network (DMN) [6]. Yao et al. reported a significant correlation between cingulate gyrus and hopelessness, cognitive disturbance and retardation in MDD patients [2], and the cingulate gyrus was inferred to play a role in the modulation of memory by emotionally arousing stimuli [46]. Besides, the superior temporal gyrus exhibited weaker responses to effort-based decision in MDD patients in a recent study [47]. In a word, these regions showed the vulnerability to the MDD, and could be served as the trait markers for MDD.

Compared to rMDD patients, cMDD patients showed significantly abnormal HE value in middle frontal gyrus, inferior parietal lobule, middle occipital gyrus and superior parietal cortex, suggesting that these brain regions may be the state markers for MDD. The middle frontal gyrus and inferior parietal lobule belong to DMN, which supports the self-reflective process, rumination and brooding in depressive patients [48, 49]. The abnormal fluctuations of both regions could add a complementary evidence for the abnormalities of DMN in MDD patients. The middle occipital gyrus belongs to visual recognition circuit [4], and several previous studies reported aberrant functional activities of middle occipital gyrus in MDD patients [4, 50]. In addition, an altered superior parietal cortex–caudate correlation pattern was reported in MDD patients in a previous study [51]. All these evidences lead to the conclusion that the depressive state is associated with the functions of these brain regions.

The current study had the following limitations. First, although much information has been acquired through HE analysis, the HE cannot provide a complete understanding of the neurobiology of depressive disorders. Other measures of complexity are needed for further understanding the mechanisms of MDD in future studies. Second, a relatively small dataset was utilized to estimate the classification accuracy. In the next step, we will collect a larger dataset and incorporate more imaging features to evaluate and improve our method.

## MATERIALS AND METHODS

### Subjects

Participants included 19 cMDD patients, 19 rMDD patients and 19 healthy subjects, and all subjects were females. The cMDD and rMDD patients were confirmed by two expert psychiatrists using the Structured Clinical Interview for DSM-IV (SCID). Totally, the cMDD and rMDD patients were different in their clinical symptoms: the cMDD patients showed depressive state at present while the rMDD patients displayed remitted mental health currently but had past histories as MDD. All depressive patients were rated on 17-item HAMD on the day of scanning. The rMDD patients were defined with HAMD scored no more than 7, while the cMDD patients scored no less than 17 [42, 52]. Inclusion criteria for the MDD patients were as follows: (1) meeting the SCID for MDD; (2) between 18 and 65 years old; (3) no history or complication of other psychiatric disorders; (4) able to give voluntary informed consent. Among the participants, one rMDD patients and three cMDD patients were medication free, and other patients were on medication including sodiumvalproate, sertraline, citalopram, lithium and divalproex. Nineteen education level and age well matched healthy subjects were recruited from the local community by print advertisements, and screened with the Non-patient Version Structured Interview from the DSM-IV. The exclusion criteria applied to all subjects includes: contraindications for MRI; with histories of stroke, neurological disorders, major physical diseases, alcohol or drug abuse, and system diseases such as thyroid dysfunction, severe anemia, syphilis or acquired immune deficiency syndrome.

After a complete description of the study to all participants, written informed consent from all subjects was obtained, and this study was approved by Research Ethics Review Board of Beijing Anding Hospital, Capital Medical University and Beijing Normal University Imaging Center for Brain Research. The procedures were carried out in accordance with the approved guidelines.

### Image acquisition

All images were collected on a Siemens 3.0 T 8 channel MRI scanner. All subjects were instructed to keep their eyes open, relaxed and awake during the scanning. To minimize head motion and instrumental noise effect, a birdcage coil fitted with foam padding was used. Resting-state fMRI data were acquired by gradient echo-planar imaging (EPI) sequence with the following parameters: repetition time (TR) = 2000 ms, echo time (TE) = 30 ms, flip angle (FA) = 90°, slice thickness= 4 mm, slices number = 33, field of view (FOV) = 220 × 220 mm^2^, matrix size = 64 × 64, and the scan lasted for 8 minutes and 240 volumes were acquired. The structural T1-weighted images were acquired without gap, and TR = 2530 ms, TE = 3.39 ms, FA= 7°, slice thickness = 1.33 mm, FOV = 256 × 256 mm^2^, and voxel dimension = 1 × 1 × 1.33 mm^3^.

### Data preprocessing

Data preprocessing were performed via the statistical parametric mapping (SPM8) (http://www.fil.ion.ucl.ac.uk/spm) and Data Processing Assistant for Resting State fMRI (DPARSF) (http://www.restfmri.net/forum/DPARSF). The first 10 images were discarded in order to make the subjects adapted to the environment and the scanner to be stabilized. At first, the remaining 230 volumes were corrected for the different acquisition time between slices. Then, all images were realigned to the first image to correct inter-scan head motions, and all subjects were included with the displacements less than 2 mm in the x, y, z axis or the angular motion less than 2°. The spurious covariates including the signals from the ventricular system, white matter and the six head motion parameters obtained from the rigid-body transform were regressed. Then, a temporal band-pass filter (0.01–0.10Hz) was applied to the time series to reduce the influences of respiratory and cardiac noise and the linear drift. Next, all resulting images were normalized to the Montreal Neurological Institute (MNI) template, and every voxel was re-sampled to 3 × 3 × 3 mm^3^. At last, all images were smoothed using a 4 mm full width at half maximum (FWHM) Gaussian kernel.

### Estimation of HE

Rescaled Range analysis, i.e. R/S analysis, can effectively detect the temporal complexity of a time series. The detailed principle of R/S analysis is listed as follows: given a time series *X* and its length is *M*, the time series is divided into *A* intervals and the length of each interval is *N* (1 ≤ *N* ≤ A), A × *N = M* . The a-th interval is marked with *I_a_* and the k-th element in *I_a_* is marked with x_a, k,_ *k* = 1, 2, 3... *N*, and *e_a_* is the average value in *I_a_* interval, then

$$y_{a,k}=\overset{k}{\underset{i=1}{\Sigma }}(x_{a,i}-e_{a}),k=1,2......N$$ (1)

$$R_{a}=\underset{1\leq k\leq N}{\max}\{y_{a,k}\}-\underset{1\leq k\leq N}{\min}\{y_{a,k}\}$$ (2)

$$S_{a}={\left[\frac{1}{N}\overset{N}{\underset{i=1}{\Sigma }}{\left(x_{a,i}-e_{a}\right)}^{2}\right]}^{\frac{1}{2}}$$ (3)

$${\left(R/S\right)}_{N}=\frac{1}{A}\overset{N}{\underset{a=1}{\Sigma }}(R_{a}/S_{a})=cN^{H}$$ (4)

Where *c* is a constant, and HE was defined as the slope of the line that fits the pairs (lnN,ln(RS)N) in a least-square sense.

### Feature selection

**Figure 6:**
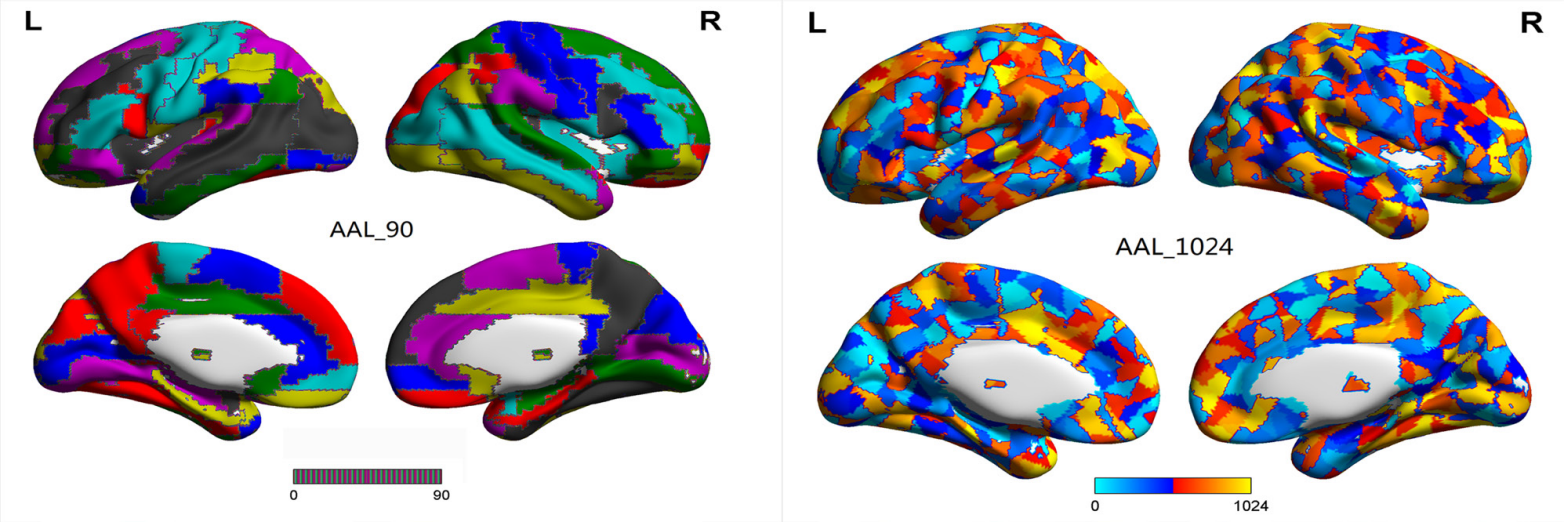
AAL-90 atlas and AAL-1024 atlas

The individual HE maps were partitioned into 1024 brain regions and 90 brain regions according to AAL-1024 and AAL-90 atlases (Figure 6). After that, the mean HE values in each brain region corresponding to two kinds of AAL atlases were served as the candidate features, respectively. Given that some features are redundant and irrelevant for classification, selecting out the discriminative features will improve the final classification performance [53, 54]. Therefore, two-sample two-tailed t tests were performed on the mean HE values of every brain region in two AAL atlases to determine the significant between-group differences as the classification features, and the significance level was set at P < 0.05. It is worth noting that the feature selection was only performed on the training set of every LOOCV fold, which could reduce the overfitting of the classifier.

### SVM based method

In this paper, a SVM based method was proposed to discriminate every pair of the cMDD patients, rMDD patients and HC groups. The SVM algorithms were proposed to find the hyperplane with maximum margin in the feature space. The minimal distance from the closest training samples to the separating hyperplane is called margin, and a larger margin corresponds to a better generalization. The training examples that lie on the margin are called support vectors, which determine the location of hyperplane and could be regarded as the most difficult data to classify. In this paper, LibSVM toolbox (http://www.csie.ntu.edu.tw/~cjlin/libsvm) was used for SVM implementation.

The proposed method adopted a radial basis function (RBF) which is defined as $(X,X_{i})\to K(X,X_{i})=e^{\gamma |X-X_{i}{|}^{2}}$ as the kernel function, and a grid search method to optimize two parameters: γ, the width of the RBF, and *C*, an input parameter for the SVM, which adjusts the trade-off between having zero training errors and allowing misclassifications. The grid search method is referred to as the classification performed with (γ, *C*) varying along a grid with $\gamma ={\text{2}}^{\text{-8}},2^{-7.75},2^{-7.5},...,2^{8}$ and $C={\text{2}}^{\text{-8}},2^{-7.75},2^{-7.5},...,2^{8}$. The input form for SVM is < *x, y* > where *x* represents classification features and *y* stands for the class labels. Here, *x* represents the mean HE values in the brain regions that showed significant differences between groups, and *y* is the predefined group label (1 or -1) in discriminating every pair of three groups. Classification performance was quantified with accuracy, sensitivity and specificity using LOOCV, and the pair (γ, *C*) with the highest accuracy rate on the 65 × 65 grid will be selected as the optimal parameters.

### Permutation test

Permutation test was used to estimate whether the observed classification accuracy was statistically significant. All class labels were randomly permuted to form a new training set, and then the feature selection and the proposed classification method were performed on the new training set. The permutation test was repeated 1000 times, and the 1000 classification accuracies formed the permutation distribution. The *P* value was calculated as the proportion of the classification accuracy with the randomized label no less than the accuracy with the original label. If *P* value was less than 0.05, it demonstrated that the actual accuracy was statistically significant.

## SUPPLEMENTARY MATERIALS FIGURES AND TABLES



## Abbreviations

MDD: major depressive disorder
rMDD: remitted major depressive disorder
cMDD: current major depressive disorder
HC: healthy controls
MRI: Magnetic resonance imaging
ReHo: regional homogeneity
ALFF: amplitude of low-frequency fluctuation
fALFF: fractional amplitude of low-frequency fluctuation
fMRI: functional Magnetic resonance imaging
HE: Hurst exponent
SVM: support vector machine
AAL: Automated Anatomical Labeling
BOLD: blood oxygen level-dependent.
RBF: radial basis function
LOOCV: leave-one-out cross-validation
HAMD: Hamilton Depression Rating Scale
ROC: receiver operating characteristics
AUCs: area under the curves
VBM: voxel-based morphometry
DMN: default mode network
SCID: Structured Clinical Interview for DSM-IV
EPI: echo-planar imaging
TR: repetition time
TE: echo time
FA: flip angle
FOV: field of view
SPM: statistical parametric mapping
DPARSF: Data Processing Assistant for Resting State fMRI
MNI: Montreal Neurological Institute
FWMH: full width at half maximum
