# Supplementary material for: Identifying current and remitted major depressive disorder with the Hurst exponent: a comparative study on two automated anatomical labeling atlases
Source: Oncotarget. 2017 Aug 3;8(52):90452–64. doi: 10.18632/oncotarget.19860 (PMC5685765; doi:10.18632/oncotarget.19860)
Supplement: Supplementary file 1 [file oncotarget-08-90452-s001.pdf]

Identifying current and remitted major depressive disorder with the Hurst exponent: a comparative study on two automated anatomical labeling atlases

SUPPLEMENTARY MATERIALS

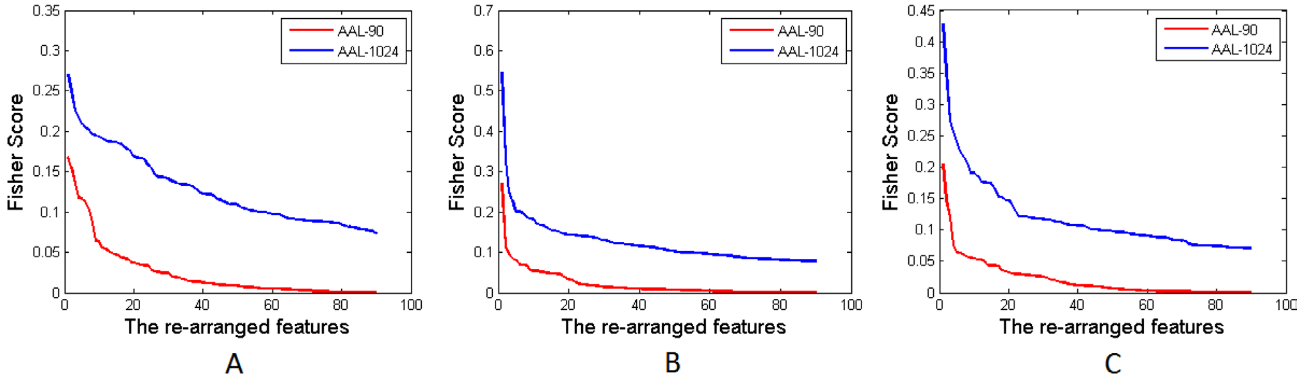

Supplementary Figure 1: The Fisher score values of the rearranged 90 features from AAL-90 atlas and the prior 90 features from AAL-1024 atlas. (**A**) between cMDD and HC, (**B**) between rMDD and HC, (**C**) between cMDD and rMDD

Supplementary Table 1: The stable discriminative features between cMDD and HC groups (appearance times > 20 in LOOCV)

AAL-90 atlas	AAL-1024 atlas
19 Supp_Motor_Area_L	1 Precentral_L
20 Supp_Motor_Area_R	**3 Frontal_Sup_L**
29 Insula_L	4 Frontal_Sup_R
33 Cingulum_Mid_L	**8 Frontal_Mid_R**
34 Cingulum_Mid_R	**9 Frontal_Mid_R**
60 Parietal_Sup_R	11 Frontal_Inf_Oper_L
81 Temporal_Sup_L	15 Frontal_Inf_Orb_L
85 Temporal_Mid_L	17 Rolandic_Oper_L
	18 Rolandic_Oper_R
	**19 Supp_Motor_Area_L**
	**20 Supp_Motor_Area_R**
	**23 Frontal_Sup_Medial_L**
	**24 Frontal_Sup_Medial_R**
	26 Frontal_Mid_Orb_R
	**29 Insula_L**
	**30 Insula_R**
	**32 Cingulum_Ant_R**
	**33 Cingulum_Mid_L**
	**34 Cingulum_Mid_R**
	36 Cingulum_Post_R
	43 Calcarine_L
	**48 Lingual_R**
	52 Occipital_Mid_R
	**56 Fusiform_R**
	57 Postcentral_L
	58 Postcentral_R
	59 Parietal_Sup_L
	**60 Parietal_Sup_R**
	**61 Parietal_Inf_L**
	**62 Parietal_Inf_R**
	**67 Precuneus_L**
	**68 Precuneus_R**
	74 Putamen_R
	**81 Temporal_Sup_L**
	82 Temporal_Sup_R
	**85 Temporal_Mid_L**

Note: The numbers in the table stand for the ID in AAL-90 atlas, and all brain regions are reported with AAL-90 atlas. Bold text indicates that more than one subregion in these regions have been reported with AAL-1024 atlas.

Supplementary Table 2: The stable discriminative features between rMDD and HC groups (appearance times > 20 in LOOCV)

AAL-90 atlas	AAL-1024 atlas
3 Frontal_Sup_L	1 Precentral_L
29 Insula_L	2 Precentral_R
30 Insula_R	**3 Frontal_Sup_L**
36 Cingulum_Post_R	**6 Frontal_Sup_Orb_R**
81 Temporal_Sup_L	**7 Frontal_Mid_L**
	8 Frontal_Mid_R
	11 Frontal_Inf_Oper_L
	13 Frontal_Inf_Tri_L
	18 Rolandic_Oper_R
	23 Frontal_Sup_Medial_L
	24 Frontal_Sup_Medial_R
	**26 Frontal_Mid_Orb_R**
	**29 Insula_L**
	**30 Insula_R**
	33 Cingulum_Mid_L
	34 Cingulum_Mid_R
	**36 Cingulum_Post_R**
	**40 ParaHippocampal_R**
	44 Calcarine_R
	45 Cuneus_L
	46 Cuneus_R
	50 Occipital_Sup_R
	51 Occipital_Mid_L
	**52 Occipital_Mid_R**
	59 Parietal_Sup_L
	63 SupraMarginal_L
	64 SupraMarginal_R
	65 Angular_L
	67 Precuneus_L
	**68 Precuneus_R**
	**81 Temporal_Sup_L**
	82 Temporal_Sup_R
	**85 Temporal_Mid_L**
	**86 Temporal_Mid_R**
	**88 Temporal_Pole_Mid_R**

Note: The numbers in the table stand for the ID in AAL-90 atlas, and all brain regions are reported with AAL-90 atlas. Bold text indicates that more than one subregion in these regions have been reported with AAL-1024 atlas.

Supplementary Table 3: The stable discriminative features between cMDD and rMDD groups (appearance times > 20 in LOOCV)

AAL-90 atlas	AAL-1024 atlas
60 Parietal_Sup_R	3 Frontal_Sup_L
62 Parietal_Inf_R	**7 Frontal_Mid_L**
70 Paracentral_Lobule_R	**8 Frontal_Mid_R**
	9 Frontal_Mid_Orb_L
	10 Frontal_Mid_Orb_R
	11 Frontal_Inf_Oper_L
	**13 Frontal_Inf_Tri_L**
	15 Frontal_Inf_Orb_L
	26 Frontal_Mid_Orb_R
	29 Insula_L
	31 Cingulum_Ant_L
	32 Cingulum_Ant_R
	34 Cingulum_Mid_R
	43 Calcarine_L
	45 Cuneus_L
	**50 Occipital_Sup_R**
	**51 Occipital_Mid_L**
	**52 Occipital_Mid_R**
	53 Occipital_Inf_L
	**60 Parietal_Sup_R**
	**62 Parietal_Inf_R**
	63 SupraMarginal_L
	67 Precuneus_L
	**68 Precuneus_R**
	**70 Paracentral_Lobule_R**
	80 Heschl_R
	**85 Temporal_Mid_L**
	**86 Temporal_Mid_R**
	**90 Temporal_Inf_R**

Note: The numbers in the table stand for the ID in AAL-90 atlas, and all brain regions are reported with AAL-90 atlas. Bold text indicates that more than one subregion in these regions have been reported with AAL-1024 atlas.
